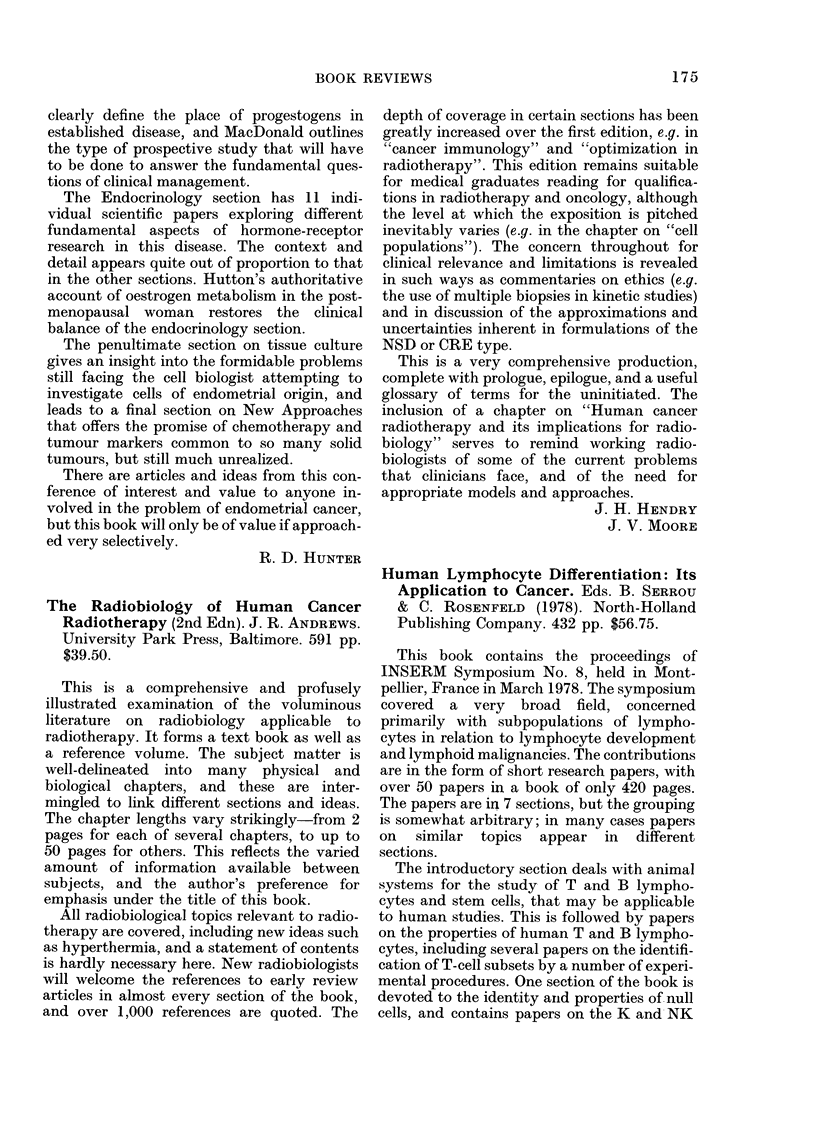# The Radiobiology of Human Cancer Radiotherapy

**Published:** 1979-07

**Authors:** J. H. Hendry, J. V. Moore


**The Radiobiology of Human Cancer Radiotherapy** (2nd Edn). J. R. Andrews. University Park Press, Baltimore. 591 pp. $39.50.

This is a comprehensive and profusely illustrated examination of the voluminous literature on radiobiology applicable to radiotherapy. It forms a text book as well as a reference volume. The subject matter is well-delineated into many physical and biological chapters, and these are intermingled to link different sections and ideas. The chapter lengths vary strikingly—from 2 pages for each of several chapters, to up to 50 pages for others. This reflects the varied amount of information available between subjects, and the author's preference for emphasis under the title of this book.

All radiobiological topics relevant to radiotherapy are covered, including new ideas such as hyperthermia, and a statement of contents is hardly necessary here. New radiobiologists will welcome the references to early review articles in almost every section of the book, and over 1,000 references are quoted. The depth of coverage in certain sections has been greatly increased over the first edition, *e.g.* in "cancer immunology" and "optimization in radiotherapy". This edition remains suitable for medical graduates reading for qualifications in radiotherapy and oncology, although the level at which the exposition is pitched inevitably varies (*e.g.* in the chapter on "cell populations"). The concern throughout for clinical relevance and limitations is revealed in such ways as commentaries on ethics (*e.g.* the use of multiple biopsies in kinetic studies) and in discussion of the approximations and uncertainties inherent in formulations of the NSD or CRE type.

This is a very comprehensive production, complete with prologue, epilogue, and a useful glossary of terms for the uninitiated. The inclusion of a chapter on "Human cancer radiotherapy and its implications for radiobiology" serves to remind working radiobiologists of some of the current problems that clinicians face, and of the need for appropriate models and approaches.

J. H. Hendry
J. V. Moore